# Meetings of Societies

**Published:** 1951-04

**Authors:** 


### Meetings of Societies

### Bristol Medico-Chirurgical Society

*Honorary Members:*

LEMUEL MATTHEWS GRIFFITHS
SIR ISAMBARD OWEN, D.C.L., M.D.
ROBERT SHINGLETON SMITH, M.D., B.Sc. Lond.
CHARLES KING RUDGE
JAMES SWAIN, *C.B., C.B.E.,* M.D., M.S. Lond.
PATRICK WATSON WILLIAMS, M.D. Lond.
JAMES PAUL BUSH, *C.M.G. C.B.E.,* Ch.M. Brist.
W. ALEXANDER SMITH, M.B. Oxon.
GEORGE PARKER, M.D. Cantab., LL.D. Brist.
JOHN ALEXANDER NIXON, *C.M.G.,* M.D., Cantab.

*Presidents:*

Lists of Presidents were published annually until 1948. Dr. Corfied, President-elect in 1938, should have assumed office in October, 1939, but owing to the outbreak of war the Society did not meet until 1945.

1945–46 HUGH HADFIELD CARLETON, D.M. Oxon.
1946–47 HUBERT CHITTY, M.S. Lond.
1947–48 THOMAS MILLING, M.B., B.Ch. Belf.
1948–49 HENRY JAMES DREW SMYTHE, *M.C., T.D.,* M.D., M.S. Lond.
1949–50 HUGH JAMES ORR-EWING, *M.C.,* M.D. Lond.
1950–51 WILLIAM AUSTEN JACKMAN, *T.D.,* M.B., B.Ch. Brist.

The Third Meeting of the 1950–51 session was held on Wednesday, 13th December, 1950. The evening was devoted to a discussion on Geriatrics. We publish the opening papers in this issue. **"The Medical Problems of the Present Day",** by Dr. C. T. Andrews, M.D., F.R.C.P. **"Some Modern Developments",** by Dr. T. S. Wilson, M.D. **"The Problem in the Bristol Clinical Area",** by Dr. William Hughes, M.D., M.R.C.P.

The Fourth meeting of the Session was held in the University on Wednesday, 10th January, 1951, and was devoted to the "flesh-creeping" subject of **"The Medical Aspects of Atomic Bombing".** In effect the subject appeared to hold little of medical interest, the only doubtful question being whether those affected died at once or after a short interval, and treatment hardly affects the issue. We expect to publish the opening papers by Drs. Tudway and Challenger in our next issue.

The Fifth meeting of the Session was held on Wednesday, 14th February, 1951. Mr. A. Dickson Wright, M.S., F.R.C.S., delivered an address on "**The Management of Intestinal Obstruction**".

The Sixth meeting of the Session was held on Wednesday, 14th March. Dr. A. M. G. Campbell gave an account of "**Some Rare Diseases Contracted from Animals**". Dr. J. Naish described "**Eczema of the Colon**".

### Devon and Exeter Medico-Chirurgical Society

On Thursday, 21st December, 1950, Dr. Margaret Hadley Jackson gave an address on "**Vaginal Discharge**". [*Lancet*, 1951, p. 92.]

At a clinico-pathological meeting of the Society on 15th February, Mr. F. C. Durbin presented the case of an interesting tumour associated with trauma arising in the thigh of a boy aged twelve, which was first thought to be an exuberant reaction to injury but proved eventually to be a fibrosarcoma. Discussion turned on whether or no hindquarter amputation should be undertaken.

Mr. P. M. G. Russell described a maternal death of a diabetic multipara: during the puerperium she developed evidence of right heart failure and was found at autopsy to have, in addition, multiple thrombosis in the anterior pituitary. Mr. R. Wayland Smith described an unusual stomach lesion on which he had operated—a cyst-adenoma of the pylorus showing evidence of malignant change; and also an uncommon case of intestinal obstruction due to a developmental anomaly in the ileum described by Bland Sutton. All these cases were discussed from the pathological angle by Dr. G. Stewart-Smith, who also described a case of meningeal dissemination of medulloblastoma. Mr. Wayland Smith also presented a case of Brodie's disease of the breast on which he had operated, and Mr. K. P. S. Caldwell one of ureteritis cystica. In both these cases the pathology was discussed by Dr. C. P. Warren. There were many admirable colour and microphotographs prepared and shown on the screen by Mr. Dyson. Lively discussion followed the presentation of each case and more than forty members were present.

### South-Western Laryngological Association

A meeting of the Association was held at Bristol General Hospital on Saturday, 6th January, Mr. G. R. Scarff, President, in the chair. A number of unusual cases were shown and produced a lively discussion. Spontaneous empyaema of nasal septum, recurrent pharyngeal polypus, subglottic granuloma (E. W. W.); congenital fenestration of faucial pillars, laryngeal changes secondary to ovarian arrhenoblastoma, chronic laryngitis (G. R. S.); naso-pharyngeal cyst (Thornwaldt's disease), chondro-sarcoma of nasal septum, deafness due to Paget's disease (J. A. J.); cerebellar ataxia from encephalitis, sarcoma of middle ear (H. D. F.); cicatricial stenosis of naso-pharynx (D. O. C.). The next meeting will be at Birmingham on Saturday, 13th October.

### British Medical Association

### Bath, Bristol and Somerset Branch

A meeting of the Branch was held at the Royal United Hospital, Bath, on Wednesday, 7th February, 1951, at 8.30 p.m. Dr. Bradley of the Ministry of Health, gave an address on " **The Common Cold** ".

### Bristol Division

A meeting of the Bristol Division was held in the University of Bristol on Friday, 5th January, 1951. Sir Lionel Whitby, *C.V.O.*, *M.C.*, delivered a B.M.A. Lecture on " **The Common Anaemias** ".

A meeting of the Bristol Division of the British Medical Association was held on Wednesday, 21st February. Mr. A. Lawrence Abel, F.R.C.S., delivered a lecture: " **Ideas from Overseas,**" illustrated by a cinematograph film.

### West Somerset Division

At a meeting of this Division at Yeovil Hospital on Thursday, 25th January, 1951, Mr. T. Holmes Sellors, Consultant Surgeon to the Middlesex Hospital, delivered an address on " **Cardiac Surgery** ". The lecture was most interesting, was illustrated by coloured lantern slides, and was well attended.

On Thursday, 8th March, at Bridgwater, Dr. Robert Forbes (Secretary of the Medical Defence Union), spoke on " **Medical Hazards in Medical Practice** ".

Future meetings.—On Thursday, 3rd May, at Taunton: Mr. Ronald Belsey will talk on " **Recent Advances in Thoracic Surgery** ".

